# Utilization of immediate post-partum long acting reversible contraceptives and its associated factors among mothers who gave birth in Addis Ababa public hospitals, Ethiopia: An institutional based cross-sectional study

**DOI:** 10.1371/journal.pone.0280167

**Published:** 2023-08-30

**Authors:** Kihinetu Gelaye Wudineh, Sisay Desalegn, Mesafint Ewunetu, Shumiye Shiferaw

**Affiliations:** 1 Department of Midwifery, College of Medicine and Health Sciences, Bahir Dar University, Bahir Dar, Ethiopia; 2 St’ Peter Specilized Hospital, Addis Ababa, Ethiopia; Haramaya University Faculty of Health Sciences: Haramaya University College of Health and Medical Sciences, ETHIOPIA

## Abstract

**Background:**

An immediate postpartum period is a good opportunity to utilize immediate postpartum long-acting reversible contraceptives for women whom they want to delay pregnancy. Long-acting reversible contraceptive methods like intrauterine device, Jaddel and implants can improve maternal and newborn health by preventing unintended pregnancy. Despite on their advantage, evidence on its utilization and associated factors is limited in our study area.Ethiopia. This study assessed the utilization of immediate postpartum long acting reversible contraceptives and its associated factors among mothers who delivered in selected public hospitals of Addis Ababa, Ethiopia, 2022.

**Method:**

An institutional-based cross-sectional study design was conducted among 420 study participants to assess the immediate postpartum long-acting reversible contraceptive utilization and its associated factors from August 30- September 25, 2022. Systematic sampling technique was used to select study participants. Data was entered into epi-data version 4.6 and analysis was performed by using SPSS version 25. Descriptive statistics and logistic regression were used. All statistical tests were significant at P-value < 0.05.

**Result:**

A total of 417 postpartum women were participated in the study making a response rate of 99.3%. Of the total study participants, 30.7% [95% CI (26.1, 35.3)] utilized immediate postpartum family planning. Women at the age of 25–34 years (AOR = 3.228[95% CI: 1.140–9.136]), had discussion with their partners about family planning (AOR = 1.891[95% CI: 1.003, 3.565]), received counseling about immediate post-partum long acting reversible contraceptive (AOR = 3.146 [95% CI: 1.489, 6.647]), had positive attitude towards immediate post postpartum long acting reversible contraceptive (AOR = 3 [95% CI: 1.770–5.648]) were associated with utilization of immediate post-partum long acting reversible contraceptive.

**Conclusion and recommendation:**

Almost one in three women delivering in health facilities of Addis Ababa Ethiopia started using immediate post-partum long acting reversible contraceptives. Discussion about contraception with partners, getting counseling about family planning on antenatal care, attitude toward contraception and the age of women were all factors that could increase IPPLARC uptake. Healthcare providers clarify any rumors about contraceptives to assure a positive and supportive attitude to increase its uptake.

## Background

The United Nations General Assembly adopted the 2030 Agenda for Sustainable Development Goals with the emphasis on universal access to a full range of safe and reliable family planning method helps couples and individuals realize their right to decide freely and responsibly the number and spacing of their children [1]. According to World Health Organization eligibility criteria, women use a variety of reversible contraceptive methods during the immediate postpartum period.

Among the long-acting reversible contraceptive methods, IUDs and implants have a low failure rate compared with tuba ligations [2]. An immediate postpartum reversible long acting contraceptive administration to women before discharge has high impact on improvement of contraceptive utilization and prevent unintended pregnancy which also improve neonatal and maternal health [3]. World Health Organization recommends post-partum women to wait for an interval at least 2 years following a live birth before getting pregnant again for better maternal and child health outcomes [4]. Immediate post-partum intrauterine contraceptive device inserted immediately following the delivery of placenta to 48 hours of vaginal or cesarean delivery can improve maternal and new born health by preventing unintended pregnancy, closely spaced pregnancy and prevent maternal death by 30% [5,6].

Early counseling of both spouse and women starting from ANC can increase the acceptability of immediate post-partum long acting reversible contraceptive which also increase the uptake [7]. The utilization of modern contraception in sub-Saharan Africa have gradual change from 13% to 29% in 1990 to 2019, but still was lower as compared to other regions [8].

Maternal death is the most and worst tragedy death in the world which can be considered as individual tragedy and human right violation [9]. Between 40% to 57% of women reported having unprotected intercourse before the routine 6- week postpartum visit [10]. Pregnancy occurring within six months of last delivery holds 7.5 fold increased risk of induced abortion, 3.3 fold increased risk of miscarriage and 1.6 fold increased risk of stillbirth [11].

Global data in 2019 showed that 44% of reproductive age women use modern family planning and the number of women desiring modern family planning method were increased, there were 1.9 billion reproductive age women (15–49), among them 1.1 billion women need family planning and 842 million are using contraceptive methods and the rests are unmet need for family planning [12].

According to mini EDHS 2019, 41% of married women whose age between15-49 are modern contraceptive method users, but we did not get any data regarding on the overall utilization of IPPLARC From this, almost all implant and IUD users obtain their method from the public sector [13]. The neonatal and under 5 mortality was higher when the birth interval was less than 2 years [14]. The utilization of IPPLARC in Ethiopia is low due to different factors. In eastern Ethiopia among one in five women delivering in health facilities used LARC. There was limited acceptance and utilization of post-partum long acting reversible contraceptive in Ethiopia [15]. Thus, the aim of this study is to assess utilization of immediate postpartum long acting reversible contraceptive and its associated factor among mothers who gave birth in Addis Ababa public hospitals.

## Methods

### Study area and period

This study was conducted in Addis Ababa, Ethiopia from August 30, 2022 to September 25, 2022. Addis Ababa is the capital city of Ethiopia which covers an area of 526.99km2. According to the 2021 population estimation, the total population of Addis Ababa was 3,774,000 [16]. There are 12 public hospitals in Addis Ababa that can provide health care to the population. Maternal and child health services are available at all of these hospitals, including family planning, ANC, delivery and post-partum care.

### Study design and population

An institutional-based cross-sectional study was conducted to assess the utilization of immediate postpartum long-acting reversible contraceptives and their associated factors among all postpartum women who were delivered in Addis Ababa selected public hospitals within 48 hours. Women in emergency conditions either medical or pregnancy-related complications that affect the women’s ability to give consent and respond to the questions were excluded from the study.

### Sample size calculation and sampling procedure

A single population proportion formula, using the assumptions of 95% confidence level and 5% margin of error was used to estimate the sample size. Utilization of immediate postpartum long-acting reversible contraceptive 53.2% was used to calculate the sample size [17]. Substituting the above assumption in the formula, the required sample size was calculated to be:

n = (Zα/_2_)^2^xp (1-p)/d^2^; where

n = Sample size

Z a/_2_ = Confidence level at 95% = **1.96**

P = Proportion of population which is 53.2%.

d = 0.05

$$n=\frac{{\left(1.96\right)}^{2}(0.532)(0.468)}{(0.05)2}=382$$

Adding 10% non-response rate the final calculated sample size will be **420.**

All postpartum women within 48 hours of delivery before discharge from the health facility and who were eligible for at least one immediate post-partum long acting reversible contraceptive method according to 2015 medical eligibility criteria were included in the study(Category 1: A condition for which there is no restriction for the use of the contraceptive method.

Category 2: A condition where the advantages of using the method generally outweigh the theoretical risks.

Category 3: A condition where the theoretical or proven risks usually outweigh the advantages of using the method.

Category 4: A condition which represents an unacceptable health risk if the contraceptive method is used).

Simple random sampling method was used to select 4 public hospitals from the total 12 hospitals. The calculated sample size was proportionally allocated to each public hospital based on the average number of institutional deliveries per month after observing three months institutional delivery reports at the selected hospitals. A systematic random sampling technique was used to select an allocated sample of postpartum women from each selected public hospital. The calculated sampling interval (K = N/n) was 4. Therefore, the first woman was selected using a simple random sampling method and consecutive women were selected at a regular interval of the fourth postpartum women from the list of delivery registration books at selected public hospitals until the required sample size was obtained.

### Data collection tools and procedures

Data was collected by interviewer- administered questionnaire. The questionnaire was prepared according to the objectives of the study adapted from relevant literatures in the local situation of the study area in English language. Questionnaires were translated in to Amharic (the local language) by an individual who had good ability of both languages then retranslated back to English by another individual to check for any inconsistencies. Four BSC midwives and one MSC in RH and one maternity specialist were used as a data collector and supervisor respectively. Data collectors and supervisors were trained for one day by the principal investigator on the study instrument, consent form and data collection procedure.

### Operational definition

**Utilization of immediate post-partum long-acting reversible contraceptives** is defined as a postpartum woman using immediate post-partum reversible long-acting modern postpartum FP methods (intrauterine contraceptive devices, juddle and implants) immediately within 48 hours of delivery [1].

**Attitude:**—Attitude towards FP was measured using 5 attitudinal questions. Participants were asked to rate their level of agreement towards FP using a 5-point Likert scale of 1 (strongly disagree) to 5 (strongly agree). Those who scored mean and above for attitude-related items have a positive attitude and those who scored below the mean have a negative attitude [18].

### Data quality control

The quality of data was assured by proper designing and pre-testing of the questionnaires at St. Poal hospital, Addis Ababa, Ethiopia on 21 of the participants. Every day after data collection, questionnaires were reviewed and checked for completeness by the supervisor and principal investigator and the necessary feedback was offered to data collectors in the next morning and before ending all session incomplete questions were completed using precoded for controlling errors during data analysis.

### Data processing and analysis

After the data collection, the data were entered, edited and coded in EpiData 4.6,then it was exported to Statistical Package for Social Sciences (SPSS) version 25 for analysis. Further, data cleaning (editing, recoding and checking missing values) was made after exported to SPSS. Descriptive statistics were computed and presented using frequencies, proportions, graphs and tables. Binary logistic regression was used to identify factors associated with utilization of immediate post-partum long acting reversible contraceptives among post-partum women. Variables with P-value less than or equal to 0.2 were selected in to multiple logistic regression models for controlling the possible effect of confounders. The Hosmer-Lemeshow goodness of fit was checked. Finally variables which had independent association with utilization of immediate post-partum long acting reversible contraceptives were identified on the basis of AOR, with 95% CI and p-value less than 0.05.

### Ethical consideration

Ethical clearance was obtained from institutional Ethical Committee of Bahirdar University. Formal letter of cooperation was written for Addis Ababa City Administration Health Bureau, to Hospitals and permission was obtained. Written informed consent was obtained from each study subjects; each respondent was informed about the objective of the study. Any mother who was not willing to participate in the study has not been forced to participate. They were also informed that all data obtained from them would be kept confidential by using codes instead of any personal identifiers and is meant only for the purpose of the study.

## Results

### Socio demographic characteristics of the participants

The total of 417 women participated in the study making the overall response rate of 99.3%. Mean age of participants was 28.55 year ±4.33 standard deviations (SD). The participant’s age ranged from 25–34 years. The majority of study participants 311, (74.6%) were urban residents. From the total of 417 postpartum women, 371(89%) were married, 129 (30.9%) had completed secondary education. One hundred fifty (27.6%) of the study participants were housewives (Table 1).

**Table 1:** Socio-demographic characteristics of postpartum women at public hospitals in Addis Ababa, Ethiopia, 2022(n = 417).

Variables	Categories	Frequencies(n)	Percent (%)
**Age**	15–24	67	16.1
	25–34	310	74.3
	> = 35	40	9.6
**Residency**	Urban	311	74.6
	Rural	106	25.4
**Religions**	Orthodox	245	58.8
	Muslim	91	21.8
	Protestant	62	14.9
	Catholic	16	3.8
	Others	3	0.7
**Marital status**	Married	371	89.0
	Divorced	25	6.0
	Single	11	2.6
	Widowed	10	2.4
**Educational status of participants**	No formal educations	66	15.8
	primary educations	129	30.9
	secondary educations	117	28.1
	college and above	105	25.2
**Occupational status**	Daily laborer	51	12.2
	Student	18	4.3
	Governmental	103	24.7
	House wife	115	27.6
	Merchant	80	19.2
	Farmers	15	3.6
	Others(Religious fathers, fisher man)	35	8.4

### Obstetrics and maternal health service related factors

About 272, (65.2%) had a history of one to two pregnancies and about 282, (67.6%) delivered their last child within two years of the previous birth.

The majority (77%) of the participants had planned pregnancy, more than half (63.5%) gave birth vaginally and 195 (46.8%) study participants wanted to delay their next pregnancy more than 24 months in the future (Table 2).

**Table 2:** Obstetrics and maternal health service related characteristics of post postpartum mothers in Addis Ababa public hospitals, Ethiopia, 2022.

Variables	Categories	Frequencies	Percent (%)
**Do you have ANC follow up?**	Yes	386	92.6
	No	31	7.4
**Number of ANC visits**	Never had visit	31	7.4
	1–4	231	55.4
	> = 5	155	37.2
**Gravidity**	< = 2	272	65.2
	>2	145	34.8
**Mode of last delivery**	spontaneous delivery	265	63.5
	cesarean sections	103	24.7
	Instrumentals	49	11.8
**Birth outcome of the neonate?**	Alive	395	94.7
	still birth	22	5.3
	Total	417	100.0
**Inter pregnancy time intervals**	< = 24 Months	282	67.6
	25–35 Months	18	4.3
	> = 36 Months	117	28.1
**What was your pregnancy intention?**	Wanted	383	91.8
	Unwanted	34	8.2
**Did your last pregnancy was planned?**	Yes	321	77.0
	No	96	23.0
**Did you have Plan to have another child?**	Yes	311	74.6
	No	106	25.4
**When to plan to conceive next child?**	< = 24 months	222	53.2
	>24 months	195	46.8
**Number of alive child**	1	189	45.3
	2–4	219	52.5
	> = 5	9	2.2

### Family planning related factors

More than half 273, (65.5%) of the study participants were using family planning before recent delivery. Two hundred sixty four (63.3%) of women had positive attitude towards the benefits of contraceptive utilization. Respondents who had above the mean score of 3.5 had a positive attitude towards IPPLRC while those with less than the mean score were considered to have negative attitude. Of the study participants, 307 (73.6%) received counseling about IPPFP during ANC follow-up. The majority of participants 302, (84.1%) discussed FP with their husband and approximately 155 (54.9%) were both involved in contraception decision making (Table 3).

**Table 3:** Family planning related characteristic of respondents in public hospitals of Addis Ababa, Ethiopia, 2022.

Variables	Categories	Frequency	Percent (%)
Did you use FP before the recent pregnancy?	YES	273	65.5
	No	144	34.5
Counseled about family planning in ANC	Yes	307	73.6
	No	110	26.4
Do you have discussion with partner on contraceptives?	Yes	283	67.9
	No	134	32.1
If YES who is main decision maker on contraceptives?	Husband	45	15.9
	Me	83	29.3
	Both of us	155	54.8
Attitude towards benefit of contraceptive	Positive attitude	264	63.3
	Negative attitude	153	36.7

### Utilization of immediate post postpartum long acting reversible contraceptive

In this study, the prevalence of immediate postpartum long-acting reversible contraceptive utilization was 128 (30.7%). More than half (57.8%) use an implanon, 24.22% use an IUD and the rest 17.97 use jaddele.

### Factors associated with Utilization of immediate postpartum long acting reversible contraceptive methods

Using bivariable logistic regression analysis, factors affecting utilization of immediate postpartum long acting reversible contraceptive methods were discussion with partners about FP, Counseled about FP in an ANC clinic, ANC flow up, Gravidity, attitude to FP, age, residency, neonatal outcome, when to plan next pregnancy, and history of FP use.

In the multivariable logistic analysis, factors that were associated with utilization of IPPLARC were age, attitude of women to wards IPPLARC, discussion with partners about FP, and counseling about FP in ANC clinics.

In this study, mothers who had a positive attitude towards immediate postpartum long-acting reversible contraceptives were 3 times more (95% CI: 1.770–5.648) likely to utilize IPPLRC than women who had a negative attitude. Postpartum women who are in the age category of 25–34 were 3.22 times more likely (95% CI: 1.14–9.13) to utilize immediate postpartum long-acting contraceptives than those in the age category of 15–24 and above 35.

Postpartum women who had discussed contraceptive methods with their husbands were 1.891 (95% CI: 1.003–3.565) times more likely to use IPPLARC than those who had never discussed it.

Women who had been counseled about FP on ANC flow up were 3.146 times more (95% CL: 1.489, 6.641) likely to utilize IPPLRC than those who had not been counseled about FP on ante-natal care integrated service (Table 4).

**Table 4:** Bivariate and multivariate logistic regression analysis showing factors associated with immediate postpartum reversible long-acting contraceptive utilization of postpartum women in public hospitals of Addis Ababa, Ethiopia, 2022.

		IPPLR Utilization				
Variables	Categories	Used(n)	Not Used(n)	COR (95% CI)	AOR (95% CI)	P-value
Have you discussed with partners about FP?	Yes	109	174	**3.792 (2.207–6.514)**	**1.891 (1.003–3.565)**	0.049*
	No	19	115	1	1	
Counseled about FP on ANC clinics	Yes	108	156	**5.54(2.85–10.77)**	**3.146(1.489–6.647)**	0.003*
	No	20	133	1	1	
ANC flow up	Yes	117	190	3.065 (1.047–8.97)	1.413(0.391–5.107)	0.598
	No	11	99	1	1	
Gravida	< = 2	74	198	1.59(1.033–2.440)	1.595(0.975–2.610)	0.063
	>2	54	91	1	1	
Attitude to FP	Positive attitude	20	133	**4.604(2.709–7.823)**	**3.162(1.770–5.648)**	0.001**
	Negative attitude	108	156	**1**	**1**	
Age	15–24	12	56	1	1	
	25–34	103	207	**2.741(1.096–6.855)**	**3.228(1.140–9.136)**	0.027*
	>35	14	26	1.082(0.542–2.160)	1.610(0.734–3.531)	0.235
Residency	Urban	105	206	1.839(1.096–3.088)	0.686(0.383–1.230)	0.206
	Rural	23	83	1	1	
Out -come of neonate	Alive	126	269	4.684(1.078–20.346)	3.344(0.662–16.511)	0.144
	Still birth	2	20	1	1	
When plan to conceive next pregnancy	< = 24 months	56	166	1	1	
	>24 months	72	123	1.735(1.140–2.64)	0.719(0.447–1.117)	0.174
History of FP USE	Yes	94	179	1.699(1.074–2.687)	0.849(0.492–1.433)	0.522
	No	34	110	1	1	

## Discussion

This study mainly assessed the prevalence of IPPFP utilization and the associated factors among postpartum women at public hospitals in Addis Ababa, Ethiopia. The overall prevalence of immediate postpartum long-acting reversible contraceptives utilization was 30.7% [95% CI (26.1, 35.3)]. This result is lower than study finding at Jimma which was 53.2% [19]. This variation might possibly be due to differences in inclusion criteria. The inclusion criteria for the study done in Jimma were postpartum women who received counselling on IPPFP within 48 hours.Our finding is higher than studies done in Tanzania, North Showa and Sidama which were 10.5%, 21.8%, and 21.6%, respectively [17,20,21]. This variation may be due to differences in sociocultural and demographic characteristics and study setting.

The implanon was chosen by the majority of postpartum women (57.8%) followed by the IUD (24.22%) and Jaddele (17.97%). When compared to previous studies, the use of immediate postpartum IUD was higher in West Gojjam, Amhara, Ethiopia (4.02%), Gamo Gofa, southern Ethiopia (14%), and Nepal (5.4%) [8,22,23]. The discrepancy may be due to improvement in health service delivery, a difference in study period, as well as the socio-economic status of the study participants. This finding was nearly similar to study done in Addis Ababa, Ethiopia (26.6%) and Rwanda (28.6%) [24,25]. The utilization of implanone was higher than study findings of Harare, Eastern Ethiopia, and the discrepancy might be due to the socio-economic differences of the study participants. But the result of this study was lower than the finding of Jimma (78%) and in North Showa, Ethiopia (81%) [17,26].

Immediate postpartum long acting reversible contraceptive utilization was significantly associated with factors such as participant age, women’s attitude towards IPPLARC, discussion with partners about FP, and counseling about FP in ANC clinics. According to the findings of this study, women aged 25–34 were 3.2 times more likely to use immediate postpartum long-acting reversible contraceptives than women aged 15–24 years. This study finding is in line with studies done in India, West Gojjam, Ethiopia, and North Showa, Ethiopia [22,27,28]. This might be explained by the fact that at this age group, mothers have awareness about the benefits of utilizing contraceptives.

This study revealed that women who had a positive attitude towards the benefit of postpartum contraceptive utilization were 3 times more likely to utilize IPPLRC than women who had a negative attitude. This study is concise with the findings of North Showa, Ethiopia [29]. This could be due to positive attitude towards certain activities being an essential factor for the actual implementation of something in real situations.

Postpartum women who had discussed contraceptive methods with their husbands were 1.89 times more likely to use IPPLARC than those who had never discussed it, which agrees with findings from Rwanda, Addis Ababa, Ethiopia, and Aroresa, southern Ethiopia [18,24,30].

The possible explanation for this might be that women can get more support to utilize maternal health services through discussion with their spouses and can also decide to limit or space their pregnancy. This might be also due to the fact that male involvement has a significant role in the use of reproductive and maternal health services. So this might increase their intention to access contraceptive methods in an efficient and timely manner after delivery.

Women who had been counseled about FP on ANC follow up were 3 times more likely to utilize IPPLRC than those who had not been counseled about FP on ante-natal care integrated services. This finding is also supported by other studies done in Rwanda, west Gojjam, Amhara, Ethiopia, Gamo Gofa, southern, Ethiopia, Harare, and Jimma, Oromia, Ethiopia [15,17,24,31,32].

The possible reason is that the fact that early starting counseling from preconceptions to ANC service increases the knowledge and changes the misunderstanding and breaks myths. In general, counseling increases the knowledge and uptake of family planning after delivery.

### Limitation of the study

This study was institutional-based and the respondents were immediate postpartum women. Therefore, the study findings are difficult to generalize to all reproductive age women in the community. Due to the cross-sectional study design, the cause and effect relations were not seen.

## Conclusion

➢ According to this study finding, almost one in three women delivering in health facilities of Addis Ababa Ethiopia started using immediate post-partum long acting reversible contraceptives.
➢ Discussions about contraception with partners; getting counseling about family planning on antenatal care; attitude toward contraception; and the age of the women were all factors that could increase IPPLARC uptake. Healthcare providers clarify any rumors about contraceptives to assure a positive and supportive attitude to increase its uptake. We also recommend researchers to come up with detailed findings especially on qualitative aspect.

## Supporting information

S1 File This is the SPSS data set of the study. (SAV)
